# Major Depressive Disorder in Iran: Epidemiology, Health Care Provision, Utilization, and Challenges

**DOI:** 10.34172/aim.2022.54

**Published:** 2022-05-01

**Authors:** Masoumeh Amin-Esmaeili, Behrang Shadloo, Afarin Rahimi-Movaghar, Seyed Mehdi Samimi Ardestani, Ahmad Hajebi, Shahab Khatibzadeh, Vandad Sharifi, Roya Samadi, Mohammad Taghi Yasamy, Mehran Zarghami, Farshad Farzadfar, Saeid Shahraz

**Affiliations:** ^1^Iranian National Center for Addiction Studies (INCAS), Tehran University of Medical Sciences, Tehran, Iran; ^2^Mental Health Department, Johns Hopkins Bloomberg School of Public Health, Baltimore, MD, USA; ^3^Departments of Psychiatry, Imam Hossein Hospital, Shahid Beheshti University of Medical Sciences, Tehran, Iran; ^4^Research Center for Addiction & Risky Behaviors (ReCARB), Psychiatric Department, Iran University of Medical Sciences (IUMS), Tehran, Iran; ^5^The Heller School for Social Policy and Management, Brandeis University, Waltham, Massachusetts, USA; ^6^Department of Psychiatry, School of Medicine, Tehran University of Medical Sciences, Tehran, Iran; ^7^Psychiatry and Behavioral Sciences Research Center, Department of Psychiatry, Faculty of Medicine, Mashhad University of Medical Sciences, Mashhad, Iran; ^8^Department of Psychiatry, Shahid Beheshti University of Medical Sciences, Tehran, Iran; ^9^Department of Psychiatry, School of Medicine AND Psychiatry and Behavioral Sciences Research Center, Addiction Institute, Mazandaran University of Medical Sciences, Sari, Iran; ^10^Non-Communicable Diseases Research Center, Endocrinology and Metabolism Population Sciences Institute, Tehran University of Medical Sciences, Tehran, Iran; ^11^Tufts Medical Center, Institute for Clinical Research and Health Policy, Boston, MA, USA

**Keywords:** Burden, Depression, Health equity, Mood disorder, Unmet need

## Abstract

One in eight adults in Iran is estimated to have major depressive disorder (MDD) – a leading cause of disability in the country. Many remain undiagnosed, and some receive only partial treatment. An estimated 60% of those with MDD were reported to have received no treatment during the past year. In this paper, we have critically reviewed the current health-care structure in the country along with prevailing patterns of health-care service utilization. We have addressed the role of psychiatrists, general practitioners (GPs), psychologists, and other health-care personnel in the treatment and care of patients with MDD, with an emphasis on the quality of service provision. In addition, the strengths and weaknesses of primary healthcare (PHC), the health insurance system, and inpatient care have been discussed. We have paid attention to the contextual issues such as mental health literacy, stigma, and healthcare inequity where relevant. Finally, practical recommendations have been provided to improve the quality of care for patients with MDD in Iran.

## Introduction

 Globally, major depressive disorder (MDD) is considered as one of the most common chronic conditions and is among the top five leading causes of disability.^1^ Despite the progress made in the science and knowledge regarding the diagnosis and treatment of depression, it has been estimated that only one out of five patients with MDD in high-income countries and one out of 27 patients in low- and middle-income countries receive minimally adequate treatment for depression.^2^

 MDD is considered as a major public health issue in Iran. In this paper, we present an overview of MDD in Iran and address present challenges regarding the management of depression. We provide statistics for mental health services according to the level of care provision (primary healthcare [PHC], and inpatient care). We also review the role of healthcare providers (psychiatrists, general practitioners [GPs], psychologists, and other healthcare workers) and the quality of the provided services. Contextual issues (mental health literacy, stigma, inequity, insurance and cost of services) are also highlighted. Some of the issues are MDD-specific and some are shared across all mental disorders. In the end, we offer suggestions for improving the care of patients with MDD in Iran. In doing so, we combined the results of a focused literature review with inputs provided by key mental health scholars, including the main authors of major mental health surveys in the country.

## The Country and Population

 Iran is a large country with an area of 1 648 000 km^2^ and a population of around 80 million. As a country covered by mountains, deserts and lush plains, the population is widely distributed. During the past decades, the country has experienced an unbalanced development, marked by industrialization and rapid urbanization, which has led to the uneven evolution of large metropolitan areas, leaving behind underserved marginalized neighborhoods due to large-scale migration to the cities and downward drift inside them. While 45.7% of the population lived in the cities in 1975, the figure has increased to more than 73.9% in 2016. The population growth rate, which was 2.9% in 1975, has reached 1.1% in 2016.^3^ In addition, the family structure has shifted from extended families living together to isolated families typically having one or two children. At the same time, there have been improvements in some development indicators. The literacy rate for adults has increased from 56.5% in 1975 to 85.5% in 2016,^4^ and life expectancy at birth has increased from 55.3 in 1975 to 75.9 in 2016.^3^

 Though classified by the World Bank as a high-middle income country, Iran suffers from socio-economic disparities, which is well reflected in health-related indicators. For instance, when categorizing the economic status to different quintiles, infant mortality rate ranged from 11.1 in a thousand live births in the most affluent to 25.1 in a thousand live births in the least affluent population.^5^ Iran has gone through several adversities such as an 8-year war, environmental and climate change consequences, drought and air pollution, natural disasters such as earthquakes and floods, and massive sanctions on the economy of the country and a significant increase in inflation rates. The long-term and chronic stress resulting from these adversities might have affected the mental well-being and health of the population.

## The Burden of MDD

 In Iran, MDD is the leading cause of disability among women and the second cause of disability following low back pain among men.^6^ Previous studies have assessed different epidemiological aspects of MDD in Iran. A systematic review of 44 studies, using diagnostic interviews, reported a pooled mean prevalence of 4.1% (95% confidence interval [95% CI]: 3.1–5.1) for current MDD in the general population.^7^ Recent studies have shown an increasing trend in the prevalence of MDD.^8^ In 2011, the latest national mental health household survey “IranMHS” was conducted to estimate the 12-month prevalence and severity of psychiatric disorders.^9^ IranMHS also aimed to determine the pattern of healthcare utilization and cost of services. The sample was a representative of the adult population aged 15 to 64 years. According to IranMHS, it was estimated that 12.7% of the adult population had suffered from MDD in the past twelve months, using a structured diagnostic interview (Composite International Diagnostic Interview, CIDI v2.1), and it was significantly higher in women than men (15.4% versus 10.2%).^9^ For this paper, we have included the results of a secondary analysis of IranMHS data on MDD. The sociodemographic characteristics of MDD are presented in Table 1. All the results provided in Table 1 are weighted based on complex sample survey analyses, which was a joint product of inverse probability of unit selection, non-response weights and post-stratification weights. The details of weighting procedure are described elsewhere.^10^ According to this study, the prototype of MDD in Iran was a middle-aged woman, residing in urban areas with low socioeconomic status.^11^

**Table 1:** Socio-demographic Factors Associated with Major Depressive Disorder^*^

**Variables**	**n**	**Weighted (%)**	**Unadjusted Odds Ratio**	**AOR (95% CI)**	***P*** ** Value**
Gender					
Male (n = 3,64)	336	10.2	1	1	< 0.001
Female (n = 4468)	678	15.4	1.61	1.85 (1.46–2.33)	
Age group (y)					
15-19 (n = 995)	106	11.2	1	1	—
20-29 (n = 2535)	369	13.7	1.27	1.34 (0.96–1.86)	0.086
30-39 (n = 2179)	260	11.8	1.06	1.07 (0.73–1.56)	0.733
40-49 (n = 1178)	162	14.2	1.31	1.32 (0.89–1.96)	0.168
50-59 (n = 697)	96	12.5	1.13	1.12 (0.70–1.79)	0.645
60-64 (n = 245)	21	10.4	0.92	0.83 (0.39–1.75)	0.624
Marital status					
Never married (n = 2019)	252	12.0	1	1	—
Married (n = 5483)	689	12.5	1.05	1.12 (0.87–1.45)	0.368
Previously married (n = 328)	73	23.6	2.26	2.17 (1.43–3.29)	< 0.001
Education					
Illiterate (n = 636)	84	12.5	1	1	—
Elementary school (n = 1901)	259	14.2	1.16	1.29 (0.92–1.82)	0.139
Middle school (n = 1269)	173	13.5	1.09	1.31 (0.90–1.90)	0.153
High school (n = 2814)	367	12.6	1.01	1.18 (0.82–1.69)	0.383
University (n = 1200)	130	11.0	0.87	0.89 (0.57–1.39)	0.609
Occupation					
Employed (n = 2785)	300	11.0	1	1	—
Students (n = 936)	111	11.6	1.06	1.15 (0.78–1.68)	0.481
Retired (n = 165)	18	9.4	0.85	0.85 (0.44–1.65)	0.626
Homemaker (n = 3219)	465	14.9	1.39	0.80 (0.62–1.03)	0.089
Unemployed (n = 726)	119	16.5	1.60	1.52 (1.13–2.04)	0.006
Residence					
Rural (n = 3484)	410	11.0	1	1	< 0.001
Urban (n = 4348)	604	13.4	1.26	1.53 (1.25–1.87)	
Socio-economic status					
Low (n = 2146)	299	14.8	1	1	—
Moderate (n = 3181)	418	12.8	0.84	0.77 (0.62–0.96)	0.020
High (n = 2323)	275	11.7	0.76	0.70 (0.54–0.91)	0.007

AOR (95% CI): Adjusted odds ratio with 95% confidence intervals. The Odds Ratio for each variable was adjusted using multivariate logistic regression model including all other variables in the table. All the percentages are weighted using complex sample survey analyses with provinces as strata and blocks as clusters., and the numbers do not necessarily match weighted percentages. Source: *Rahimi-Movaghar et al.^11^

 MDD is associated with significant functional impairment and negative consequences. More than one-fourth (26.7%) of the participants with MDD reported severe functional impairment; 39.8% and 33.5% reported moderate and mild impairment, respectively. Suicide attempt was reported in 5.4% (95% CI: 4.0-7.2) of those with the diagnosis of MDD in the preceding year. MDD has also been reported in 51.2% (95% CI: 39.7-62.5) of all suicide attempts.^11^ A systematic review has reported that 45% of suicide attempts were associated with depressive disorders.^12^

## National Policies

 In the past two decades, the Ministry of Health (MoH) has fully acknowledged the clinical and public health importance of MDD. Several national programs, such as “National action plan for non-communicable diseases prevention and control”, “National mental health priorities”, “Integration of diagnosis and treatment of mental disorders in PHC based on the mhGAP Intervention Guide”, and “Community Mental Health Centers” (CMHCs) have paid significant attention to public awareness, advocacy, diagnosis and treatment of depression.^13-17^ Despite growing attention to the identification and management of patients with MDD, advocacy and resource allocation for this condition are not as great as those for other competing priorities such as maternal healthcare or diabetes and hypertension.

## Healthcare Provision for Patients with MDD

 Approximately two-thirds of the services for mental disorders are provided by the private sector and the rest by the public sector.^18^ Publicly funded PHC system provides essential healthcare services; however, the utilization of PHC services is limited in urban areas. More than half of those affected by mental disorders receive outpatient mental health services from GPs practicing in their private offices or as a part of the PHC system. One-third of the mental care visits are provided by psychiatrists.^18^

## General Practitioners

 The country benefits from a high number of GPs. By 2017, there were more than 92 000 GPs in the country.^19^ As mentioned earlier, mental health services are mostly provided by GPs. According to the IranMHS data, 43.5% of those with MDD who received any health service had been visited by a GP at least once during the past 12 months, which is very close to the corresponding rates for other mental disorders.^11^ GPs receive training for the detection and treatment of mental disorders during their one-month clerkship and one-month internship psychiatry rotations. There have also been additional in-service training courses for GPs within the national primary health care system which covers the entire rural and most of the urban areas.^20,21^

 However, mental health services provided by GPs are associated with some challenges. Most of the pre-service training programs are conducted in tertiary settings where common mental disorders are not frequently seen, resulting in lower than optimal knowledge and skills for the management of depressive and anxiety disorders. Compared to the general medical conditions, GPs seem to feel less competent and interested in the management and care of mental disorders when they graduate. In addition, due to the usual high workload in both public and private sectors, GPs face significant limitations in allocating adequate time for efficient psychiatric interviews, necessary for the detection and diagnosis of mental disorders. At least in the private sector, there exists almost no planned surveillance system to monitor the quality of services that are delivered by GPs for patients with mental disorders. GPs working outside the PHC system are unaware of the existing guidelines and therefore, rarely abide by the common clinical guidelines. The insurance system also does not acknowledge the existing guidelines and does not use them for auditing the treatment process.

 In recent years, a collaborative care model through CMHCs has been introduced. CMHCs, led by a psychiatrist, mainly focus on diagnosis, pharmacological treatment, and provision of psychoeducation for common mental disorders, i.e. depressive and anxiety disorders according to their specific guidelines. Through this collaborative model, GPs practicing in private sectors or public urban healthcare centers within a specific catchment area are linked to a CMHC and receive training and supervision, as well as bi-directional referrals.^17^ Evaluation of the program showed an increasing rate in the identification and treatment of patients with MDD or anxiety disorders. Given its promising results, the MoH is gradually expanding this model particularly in urban areas throughout the country.^13,22,23^

## Psychiatrists

 In 2018, there were about 1756 psychiatrists (2.2 psychiatrists per 100 000 population) in the country. The number has doubled in the last decade (personal communication with the Iranian Psychiatric Association). Although this proportion is higher than the global average (1.3 per 100 000) and the Eastern Mediterranean region (1.2 per 100 000),^24^ the distribution of psychiatrists is geographically uneven, varying from 0.4 to 7.5 psychiatrist per 100 000 population in different regions.

 Iran benefits from extended psychiatry training programs. Twenty-four universities of medical sciences provide psychiatry residency training programs. There are five subspecialized child and adolescent psychiatry training and seven psychiatric fellowship programs in the country.^19^ Since 2009, the duration of training for residency program has increased from three to four years, with extended training on community-based programs and non-pharmacological treatments.

 Though psychiatrists are competent in the diagnosis and treatment of MDD, their contribution to public mental health is facing several challenges and shortcomings. Due to the higher income and better living conditions, psychiatrists tend to concentrate in major cities. In some areas, patients in need of psychiatric care have to travel for hours to reach a psychiatrist. Poor interdisciplinary teamwork and insufficient non-pharmacological interventions and a high patient load of some private and public psychiatric clinics are two other notable shortcomings of patient care delivered by psychiatrists. Payers do not cover non-pharmacological interventions for psychiatric patients in the private sector and most people cannot afford psychotherapy services either. It seems that overuse of psychiatric medications, use of costly new medications, polypharmacy, and overuse of electro-convulsive treatment, and newly introduced brain stimulation techniques such as repetitive transcranial magnetic stimulation and transcranial direct current stimulation by some psychiatrists, though not yet widespread, are among other challenges of specialty-level psychiatric care in Iran. The regulatory system is inadequate. Evidence-based national guidelines or standards of care are still in the making, and ongoing training and monitoring are yet insufficient.

## Clinical Psychologists

 During the last couple of decades, there has been an unprecedented and indiscriminate increase in undergraduate and postgraduate training programs in almost all academic majors. After a long period of shortage of psychologists and counselors, currently, there are tens of thousands with a master’s degree or Ph.D. in the country. Postgraduate clinical psychologists can follow their career in the private sector through establishing their own private offices, or work in inpatient or outpatient settings in a multi-disciplinary team. They benefit from rich theoretical training, but in many cases, the programs lack proper clinical settings. Therefore, not all clinical psychologists have adequate clinical expertise for the diagnosis of mental disorders and providing different types of psychotherapy, especially cognitive behavioral therapy. In addition to clinical psychologists, there are other psychologists or counselors who become involved in clinical work without relevant training. There are yet no clinical guidelines and regulatory systems for psychologists. There is a real need to bring on board qualified clinical, health psychologists for prevention and management of clinical conditions such as depression. With the current reform in the healthcare system, clinical psychologists are now being recruited by the PHC. Their role in the PHC has been discussed under the corresponding section.

## Other Specialists

 Some patients show more willingness to receive treatment for their mental health problems by non-psychiatric medical specialists (such as neurologists, neurosurgeons, and cardiologists), mainly due to a lower sense of stigma or low mental health literacy. According to the IranMHS data, 15.2% of those with MDD who received any outpatient health services during the past 12 months had been visited by non-psychiatric medical specialists, with only one third having received adequate treatment.^11^ These specialists receive almost the same level of training for the management of psychiatric disorders as GPs, i.e. only pre-service training. As a result, the pattern of problems is the same and services might be associated with misdiagnosis, incomprehensive care, mere symptomatic management or overmedication.

## Family, Community, and Social Support

 In Iran, families are still considered as the primary caregivers for patients with psychiatric disorders. This seems to be also true for MDD. Families assist in reminding or even administering patients’ medications and have a key role in encouraging patients for follow-up visits. Moreover, a large number of families would take responsibility for the care of the patient and are reluctant to use inpatient services, especially for girls and young women, mainly due to the stigma associated with psychiatric hospitalization. This has also been reported in many other developing countries.^25^

 Except for the role of the families, the social support system is underdeveloped in Iran. There are hotlines available for emergencies, which provide services for patients with MDD, especially those with suicidal thoughts and plans. Public social services, including residential facilities, home visits or day centers, are available mainly for those suffering from specific severe and chronic psychiatric conditions (e.g. schizophrenia or severe substance use disorders). These services are mainly attached to psychiatric hospitals and are therefore merely provided to patients after discharge and do not cover all the demand. There are also a limited number of social workers involved in the care of psychiatric patients, who are mainly affiliated to psychiatric hospitals. The role of social workers in the hospitals, however, seems inadequate. They usually help patients with financial issues and in some cases provide liaison with the families. Overall, the role of social workers in the treatment and care of depression could be considered as minimal.

 There are some non-governmental organizations (NGOs), whose work mainly focuses on mental disorders. A number of them are working on substance use and few of them on chronic disorders such as schizophrenia. Other disorders, including depression, are almost absent from the map. There are no self-help groups for people with MDD.

## Primary Healthcare System

 In the public sector and mainly in rural areas, there is a multilevel provision of services, which starts from health houses, staffed with community healthcare workers, backed by rural and urban health centers. Healthcare staff is led by GPs, supported by a tertiary level of specialized care in the large cities. The PHC has had a wide range of duties including annual health screening and active case finding which includes mental health conditions, public education, immunization and disease control, family planning, and environmental health. The structure was well developed at the national level and has been operational in rural and remote areas, leading to significant improvements in the general health indicators. However, this has not been the case for the recently inflating urban areas and their suburbs.

 With the rapid growth of urbanization and a shift from communicable to non-communicable diseases, authorities decided to go through the Health Transformation Plan (HTP), formally launched in 2014. The main objectives of the HTP were universal healthcare coverage through insurance for all, and decreasing the out-of-pocket expenditure. A comprehensive program of urban healthcare delivery based on a health professional team (consisting of community healthcare worker, GP, and other health staff) benefiting from an online registry system was an important component of the HTP.

 Since the late 1980s, mental health services have been integrated into the PHC at different levels. These services include the screening of mental disorders by a minimally trained healthcare worker to treatment by the GPs ^20^ and if indicated, referral to higher levels of the health system. In the beginning, the main focus was active case finding of serious neuropsychiatric disorders (consisting of psychotic disorders, mental retardation, and epilepsy). Passive detection of common mental disorders (consisting of depressive and anxiety disorders) was the second priority. The program had been evaluated several times and showed that the short-term training of the healthcare personnel resulted in lasting improvement of their knowledge and attitude.^26^ Although the performance of the team was acceptable in detection of severe mental disorders, it was less efficient for detection of common mental disorders. It was estimated that only 0.5 to 7.6 in 1000 population were diagnosed as common mental disorders at the PHC and it was suggested that screening and diagnostic tools, training contents, and implementation methods should be improved to achieve more desirable results.^27^

 With the introduction of the HTP, the focus of the PHC program on mental health screening shifted from passive detection to active screening and case finding of common mental disorders. It aims to improve detection and care in both rural and urban areas. Since the implementation of the HTP, the mental health program in the PHC includes an integrated electronic registry system and a well-designed evidence-based national service package which supports staff with detailed assessment and management protocol. The service package includes screening for common mental disorders using Kessler Psychological Distress Scales (K6)^28^ conducted by the community healthcare worker and providing mental health services by the GP, and a trained clinical psychologist who is now a new member of the healthcare team at the urban PHC centers. Those who have been screened positive for depression are referred to GPs for further evaluation and diagnosis. GPs use a national PHC-specific protocol, which is adapted from the World Health Organization (WHO) mental health GAP action program (mhGAP), for diagnosis, treatment, and referral of patients with MDD.^29^ The referrals are supposed to be made to psychiatrists in tertiary settings. Adherence to treatment and follow-up visits are monitored by the community healthcare worker. A clinical psychologist provides at least two sessions of patient and family psychoeducation following the GP’s visit.

 Currently, mental health services in the PHC have a proper and detailed design on the paper. Nevertheless, we face challenges in practice:

- Community healthcare workers are typically midwives by training and are strictly supervised for their role in maternal and child healthcare, but overlooking their tasks and responsibilities regarding mental health services.
- The majority of the PHC service users are women of childbearing age. Women in other age groups, as well as adult men, tend to utilize the PHC services less frequently.
- Although both community healthcare workers and GPs receive an additional short-term in-service training on mental health by the PHC, they seem to have less knowledge, skill, and interest in their mandate for mental disorders compared to other medical conditions.
- The PHC staff who face high workload prefer to focus more on the physical problems of the patients than their mental health and well-being.
- There is a high turnover of GPs in the PHC and the system is reluctant to invest in long-term and continuous training for these transitory GPs.
- Confidentiality and data security are major concerns. The current measures are not sufficient to ensure the patients to share their sensitive mental health issues with ease and convenience.
- The referral and back-referral systems sometimes function improperly. The system’s bureaucracy and poor coordination between the levels are among the contributing factors. Suboptimal referrals significantly lower the quality of services and the chance for sound follow-up.

## Inpatient Care

 At the specialized care level, inpatient services are mainly provided by the public sector. In recent decades, the government’s policy has been to stop the establishment of new psychiatric hospitals and to expand the number of psychiatric beds in general hospitals. We have more than 10 000 psychiatric beds in 39 psychiatric hospitals (8.5 beds per 100 000 population) and 159 psychiatric wards in general hospitals (4.7 beds per 100 000 population). In 2016, 124 admissions per 100 000 population in psychiatric hospitals and 81 admissions per 100 000 population in psychiatric wards in general hospitals were reported.^24^ Occupation rates for psychiatric beds are quite high with waiting lists for elective admissions. There is a high demand for emergency psychiatric services while the number of psychiatric emergency beds is relatively low.

 However, the treatment of patients with MDD in hospitals has acceptable quality. An increasing number of them are receiving psychotherapy covered by insurance. Suicide prevention and management protocols, as well as guidelines for electroconvulsive treatment, are operational within the psychiatric wards. Patients with depressive disorders usually prefer to be admitted to psychiatric wards in general hospitals due to lower stigma. We believe this could result in lower stigma, more comprehensive interventions, receiving consultation-liaison psychiatric services, more interaction with other disciplines of medicine and a higher quality of services with a chance to attend to other medical comorbidities.

 There are some limitations associated with inpatient care. With the current demand, we have faced the challenge of a low number of psychiatric beds, and more importantly, a large disparity in the number of psychiatric beds and access to inpatient care in the country. However, allocating additional beds is not the best solution. We need to provide adequate active treatment follow-up and aftercare services. There is now a movement toward the community-based treatment of psychiatric disorders, especially for MDD. Collaborative care through CMHCs seems to be a feasible and effective model for Iran.

## Medications

 The pharmacopeia of Iran includes a diversity of medications for treatment of depression. New psychiatric medications, which have proven to be effective, usually receive approval from the Iranian FDA and become available to patients within a short period. The majority of medications manufactured in Iran are allowed to be marketed following bioequivalence studies, and usually have a relatively low price (one US cent per amitriptyline pill to 20 US cents per escitalopram pill). Brand medications, manufactured by companies abroad, are much more expensive. The insurance system covers the majority of the medications (especially those manufactured in the country). If covered by insurance, the patient would have to pay around 30% of the cost. Recently, the availability and price of medications, compared to other modalities of mental health services, have been affected most by several reasons such as sanctions, shortages in the supply chain and marketing policies of pharmaceutical companies.^30^

 There is a list of essential drugs at the PHC centers. The list consists of almost 30 psychiatric medications, including eight antidepressants (amitriptyline, clomipramine, imipramine, nortriptyline, trimipramine, citalopram, fluoxetine, and fluvoxamine) and several mood stabilizers, which are provided free of charge in rural health center pharmacies for psychiatric patients.

 In Iran, pharmacies do not have permission to prescribe medications and refill prescriptions without a physician’s authorization. However, it is not uncommon for patients to directly visit pharmacies demanding prescription-only medications, to get new medications or to refill their previous prescriptions. The 2011 national survey showed that a significant proportion (20.6%) of those diagnosed with MDD had sought help from pharmacists.^11^ Despite the existing regulations, monitoring and supervision are inadequate and there is no data on the nature of such pharmacy-provided services.

 Although an increasing trend of antidepressant prescriptions has been reported in the last decade,^31^ there is no published study to compare the rate of antidepressant prescriptions by psychiatrists with other professionals and GPs in Iran.

 To prevent over-prescription encouraged by the pharmaceutical industry, prudent steps were recently taken by the Iranian Psychiatric Association. According to a statute drafted by the Association’s board of directors and endorsed by the Iranian Psychiatrists General Assembly two years ago, clear boundaries were drawn with the pharmaceutical industry and guidance was provided for interactions with the industry to make it as transparent as possible and to minimize conflicts of interest.

## Service Utilization

**Figure 1:**
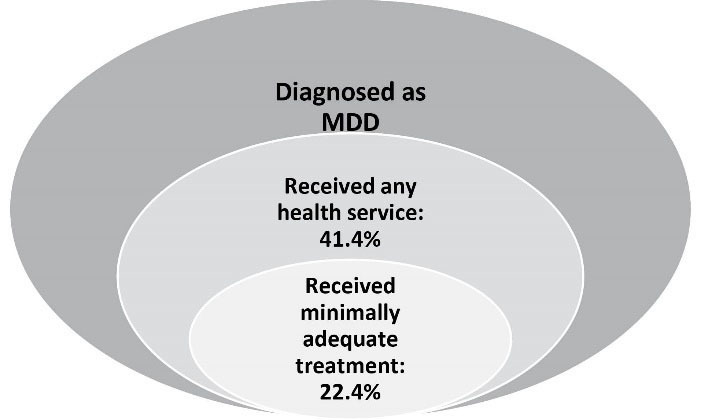
Venn Diagram of Service Use in those with MDD in the Past 12 Months. Source: Rahimi-Movaghar et al.^11^

 According to IranMHS, among those diagnosed with MDD, 76.7% (95% CI: 73.2-79.9) had perceived the need to seek treatment for their mental health problems and 41.4% (95% CI: 37.7-45.2) had utilized health services for MDD (Figure 1). In addition, a significant proportion (49.6%) of those with MMD who had received care had utilized it with more than 30-days of delay. For those suffering from MDD, the number of days between the initial perceived need to seek help and visiting a health service provider ranged widely with a median of 287.6 days. Overall, male sex, younger age, being single, having higher education, not having insurance coverage and belonging to both low and higher (compared to medium) socio-economic status were significantly associated with lower service utilization for mental health.^11^

 According to the patients, the most common reasons for under-utilization of services (either not visiting or visiting a provider with delay) were incorrect beliefs about their depression. Some patients believed that the problem was self-limiting or they could manage it themselves. Some accepted that there is no effective treatment or they were concerned about the adverse effects of medications. Along with these reasons, which were associated with low mental health literacy, the cost of services followed by the stigma of mental disorder diagnosis were the other major reasons for under-utilization of services.^18^ This is in contrast with most lower-income countries where females and not males underutilize services, possibly due to lack of access to services and limitations for women.^25^ In Iran, it is also common that families and significant others of the patients with depression confuse symptoms of depression with laziness or accuse the patient of malingering. There is also a negative attitude towards psychiatric medications and a significant proportion of the general population believes that the medications are addictive.^11^

 In the last decades, significant attention has been paid to mental health literacy and stigma of mental disorders in Iran.^32,33^ Several programs and mass media campaigns have targeted stigma and negative attitudes toward psychiatric disorders, especially MDD. In a cohort study on the youth population (Persian Youth Cohort-PYC) (http://persiancohort.com/persian-youth-cohort/) which is part of the PERSIN cohort,^34^ participants were inquired about their attitudes toward mental health service utilization. According to this study, 87% indicated that they would use a health service for their mental problems if they were affected, and 93% indicated that they would openly discuss their issues with their healthcare provider. However, 64% of the participants stated that they feel embarrassed if others find out about their referral to a mental health professional. Therefore, it seems that some aspects of stigma still act as a major obstacle for seeking professional help.^35^

 Moreover, adherence to the treatment has a major impact on the efficacy of the provided services. According to IranMHS, 64% of patients fully adhere to their medication regimen for their mental health problems. The side effects of medications have been reported as a common factor for treatment/medication cessation. Psychoeducation for both patients and families seems to be inadequate and needs further promotion. Inadequate continuity of care and active follow-up, even after discharge from inpatient settings, contributes to the low adherence.

 To picture the complete map of service utilization, it is also necessary to consider the quality of provided care. In IranMHS, a simplified definition of minimally adequate treatment was used as an indicator for the quality of services. The definition was derived from the World Mental Health Survey as either having made at least two visits in the last twelve months or having made a visit to the healthcare system for a mental health issue in the last two weeks. We found that among those suffering from MDD who had used health services in the prior twelve months, only 54.2% had received minimally adequate treatment (Figure 1).^18^

 Currently, insurance coverage is quite high in the county (more than 93%).^36^ The HTP resulted in a further expansion of insurance coverage as well as reducing the out-of-pocket cost. Now, the insurance system pays for 90% and 70% of the treatment price for inpatient and outpatient services provided by the public sector, respectively. Since the implementation of the HTP, some insurance companies cover seven non-pharmacological interventions, only if a psychiatrist provides them in the public sector. However, the insurance system’s payments for services provided by the private sector are pretty low. Considering the long-term duration of treatment, the inpatient and outpatient costs of treatment in the private sector are relatively high. On the other hand, traditionally, the general population perceives that the quality of services in the private sector is higher than the public sector. Therefore, people are more willing to visit private offices. Outpatient treatment by the private sector for depression outnumbers those utilizing the public sector: 66.5% (95% CI: 60.8–71.7) versus 50.2% (95% CI: 44.4–56.0), respectively.^11^ This results in a high financial burden on the families of the affected patients and has led to catastrophic healthcare expenditure for one-fifth of patients with MDD.^11^

## Recommendations

 As described before, MDD is one of the most common psychiatric disorders with a high burden in Iran. The treatment and care for depression need a diverse group of healthcare professionals. In the last three decades, the Ministry of Health has implemented an array of programs for the promotion of mental health. The main target was diagnosis and treatment of depression provided by healthcare professionals in various settings. Here, we aim to address the related challenges and to suggest a list of priorities to promote the coverage and quality of care for MDD.

## Mental Health Literacy and Destigmatization

 There is a consensus that mental health literacy, as a significant factor for service utilization, still has a large space for improvement. Interventions focusing on the promotion of health literacy can increase actual service utilization for depressive disorders.^37^ Attitudinal barriers in initiation and adherence to the treatment are shared across different countries around the world.^38^ A significant number of people are not aware of the nature of the disorder and the need for treatment. Depression is still associated with a high stigma, and a large proportion of people feel embarrassed to utilize psychiatric services. To change the attitude and improve mental health literacy, multiple approaches are required. For this purpose, we suggest the following interventions. Community-initiated movements for raising public awareness in which patients or their families narrate their personal experiences, mass media programs, local events at public centers, and school-based programs can be effective. In addition, we have high internet and cell phone penetration rates. Therefore, we are capable of using extensive web-based interventions, using smartphone applications, and social networks that are effective in the general population.^39-41^

 To improve the help-seeking behavior of patients and their families, we need more specific and face-to-face interventions. Motivational enhancement techniques should be among our priorities.^41^ The contribution of NGOs should be fully supported and facilitated. We also need psychoeducation after detection and initiation of treatment to improve adherence to treatment. Necessary information on pharmacological and non-pharmacological interventions and the minimum duration of treatment should be provided. Utilizing the low-intensity series of psychological interventions, developed by the WHO, may help in reducing the cost of treatment.^42^

## Inequity

 There are large disparities regarding availability, accessibility, and affordability of mental health services. The distribution of healthcare resources, specifically psychiatrists and specialized inpatient services, is not optimal. More disadvantaged areas are suffering from a higher burden of depression where specialized care is scarce. There should be alternatives for specialized psychiatric care. A former program of intensive training of GPs for detection and treatment of psychiatric disorders was launched with relative success but it was abandoned. The intensively trained GPs can act as substitutes for psychiatrists in remote areas. Another approach is higher salary and benefits for psychiatrists working in more disadvantaged areas. Telepsychiatry can also be beneficial for remote areas. Moreover, general hospitals are available even in small cities all over the country. We should allocate a number of their beds to specialized inpatient psychiatric services.

## Strengthening Mental Health in the PHC

 The integration of mental health services into the PHC system has been well designed, but poorly implemented. Now, improving the performance of the new PHC system is one of the main priorities of the different health sectors. We need continuous and in-depth training of PHC staff, to be accompanied by close supervision and monitoring to overcome most of the previously mentioned barriers and challenges. The structure of the PHC should be revisited to tackle the issues regarding referrals and back-referrals. It has been observed that CMHCs, that have been developed as a higher level of mental health-care using a collaborative care approach, can efficiently fulfill the mentioned roles, and should be scaled up. The promotion of community services along with rehabilitation could reduce the need for more intensive and costly treatments such as inpatient care. The insurance system could have a key role in the expansion of community services through endorsing community service packages.

## Guidelines and Standards of Mental Healthcare

 We do not have enough data on the extent of the implementation of guidelines for diagnosis and treatment of depression. Although IranMHS has provided data on the epidemiology and cost of services, we did not find any longitudinal study at the national level that has measured the real-time elements and continuity of care. We also do not know the cost of services during the entire episode of the disorder.

 Although a great number of national guidelines for general medical conditions have been developed, no standard of care for MDD has been developed in the country. We need standards and ongoing supervision to make sure that patients are receiving high-quality care. There are protocols for treatment of depression, designed only for GPs who are working in the PHC and for those who are collaborating with the CMHCs. However, GPs’ adherence to the protocol has never been assessed and is not being monitored. We need to develop our customized treatment guidelines urgently for each level of care and each group of healthcare providers, backed by a monitoring and surveillance system.

 The “Iran Quality of Care in Medicine Program” (IQCAMP) has been designed to assess the value of care, defined as quality per cost for medical conditions in the country. IQCAMP covers high-burden and high-cost conditions including MDD and aims to generate a series of standards for the quality of care and several indicators for measuring them. These standards include risk assessment of suicide for all, a thorough assessment of substance use disorders and bipolar spectrum, periodic examination for adverse reactions to medications, and provision of psychoeducation for both the patients and their families.

 We are also developing a service package for MDD and will then estimate the overall costs. The package includes both pharmacological and non-pharmacological treatments with a standard minimum duration of treatment. The service package can promote treatment adherence and continuity of care, and also help the payers to monitor the services.

 In conclusion, MDD has a high prevalence and is associated with a significant burden. Treatment and management of MDD require a comprehensive approach. Optimal treatment and care for MDD are within reach. We believe that implementation of the recommendations can result in a significant decrease in the burden of MDD in the country.
